# Supplementary material for: Mitigating Diabetic Cardiomyopathy: The Synergistic Potential of Sea Buckthorn and Metformin Explored via Bioinformatics and Chemoinformatics
Source: Biology (Basel). 2025 Mar 31;14(4):361. doi: 10.3390/biology14040361 (PMC12024933; doi:10.3390/biology14040361)

Supplementary Figure S1

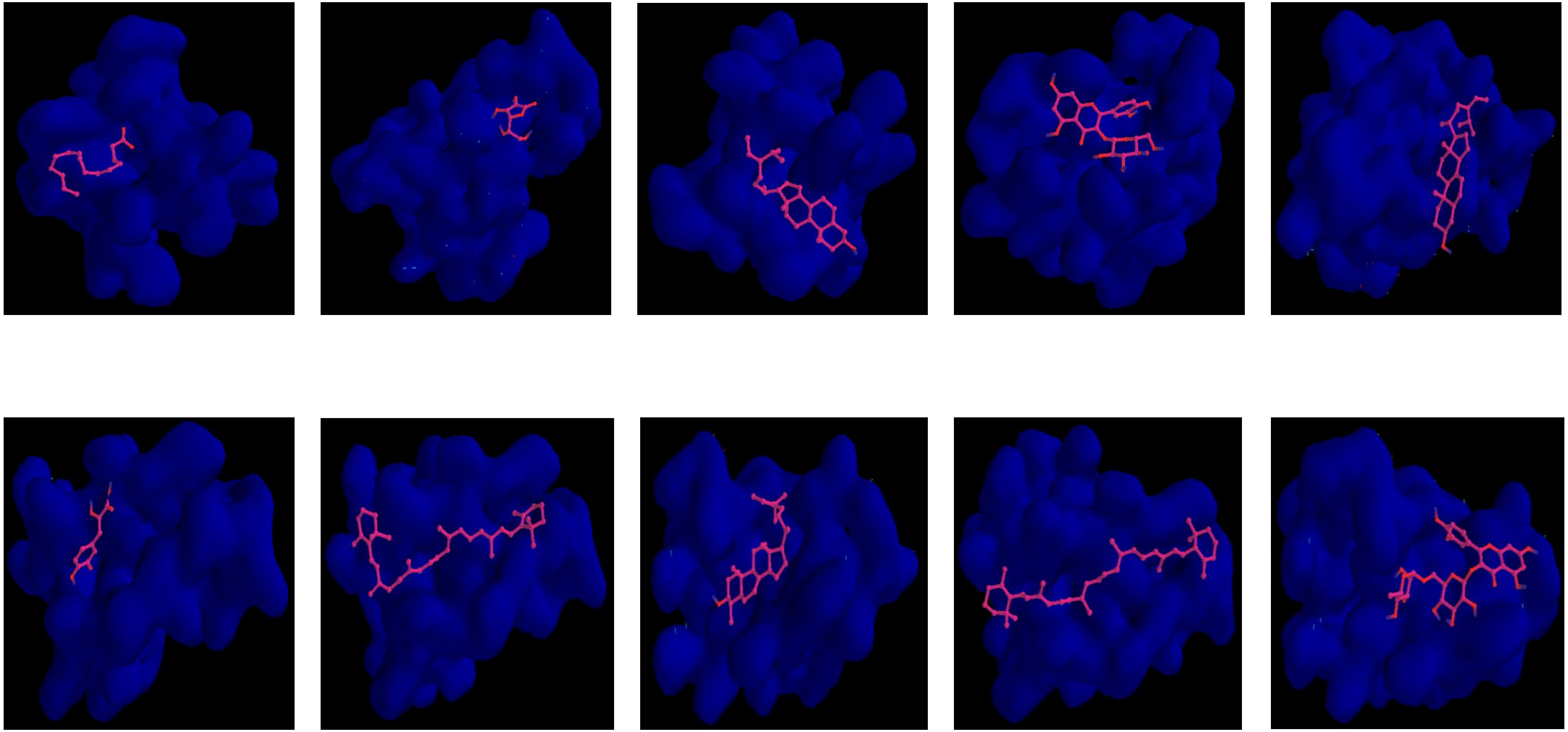

Supplementary Figure S1

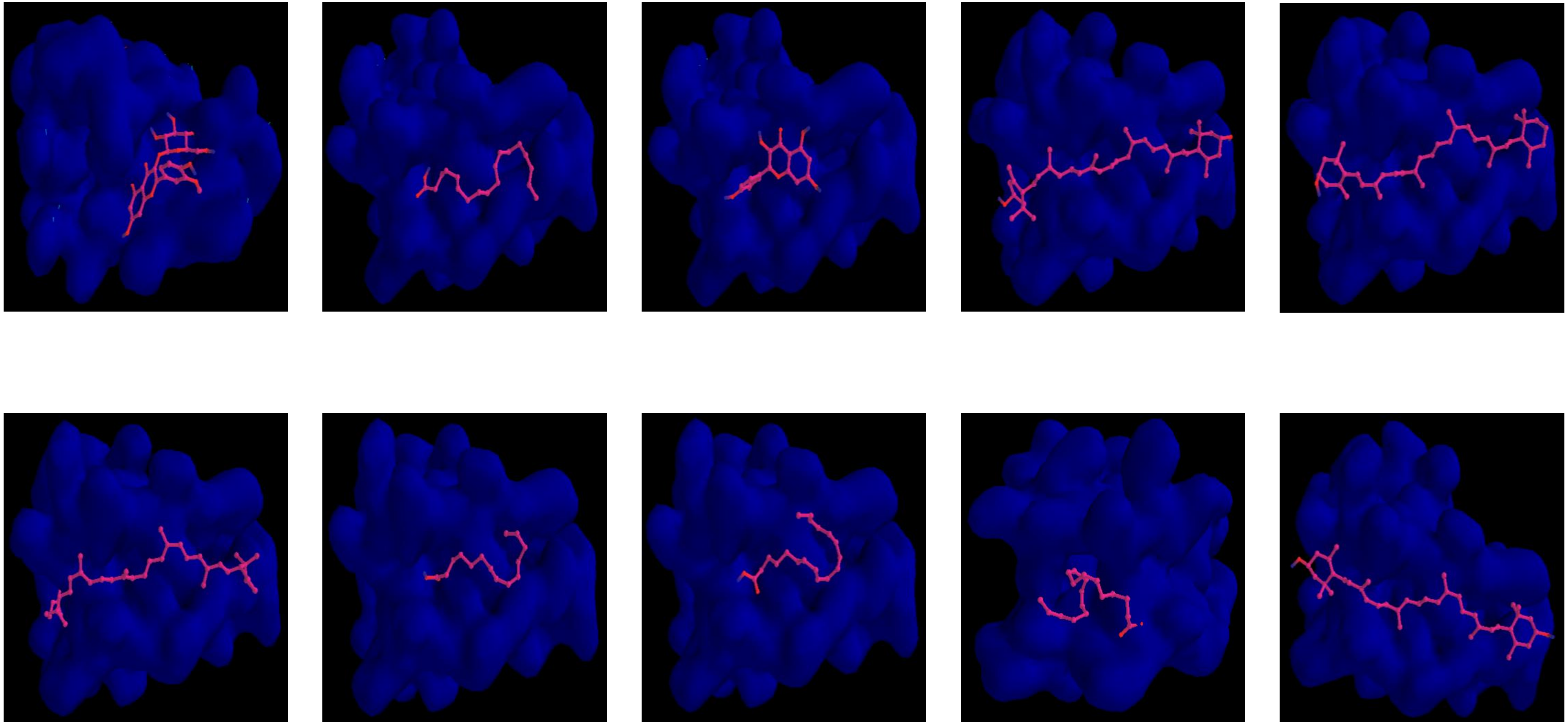

Supplementary Figure S1

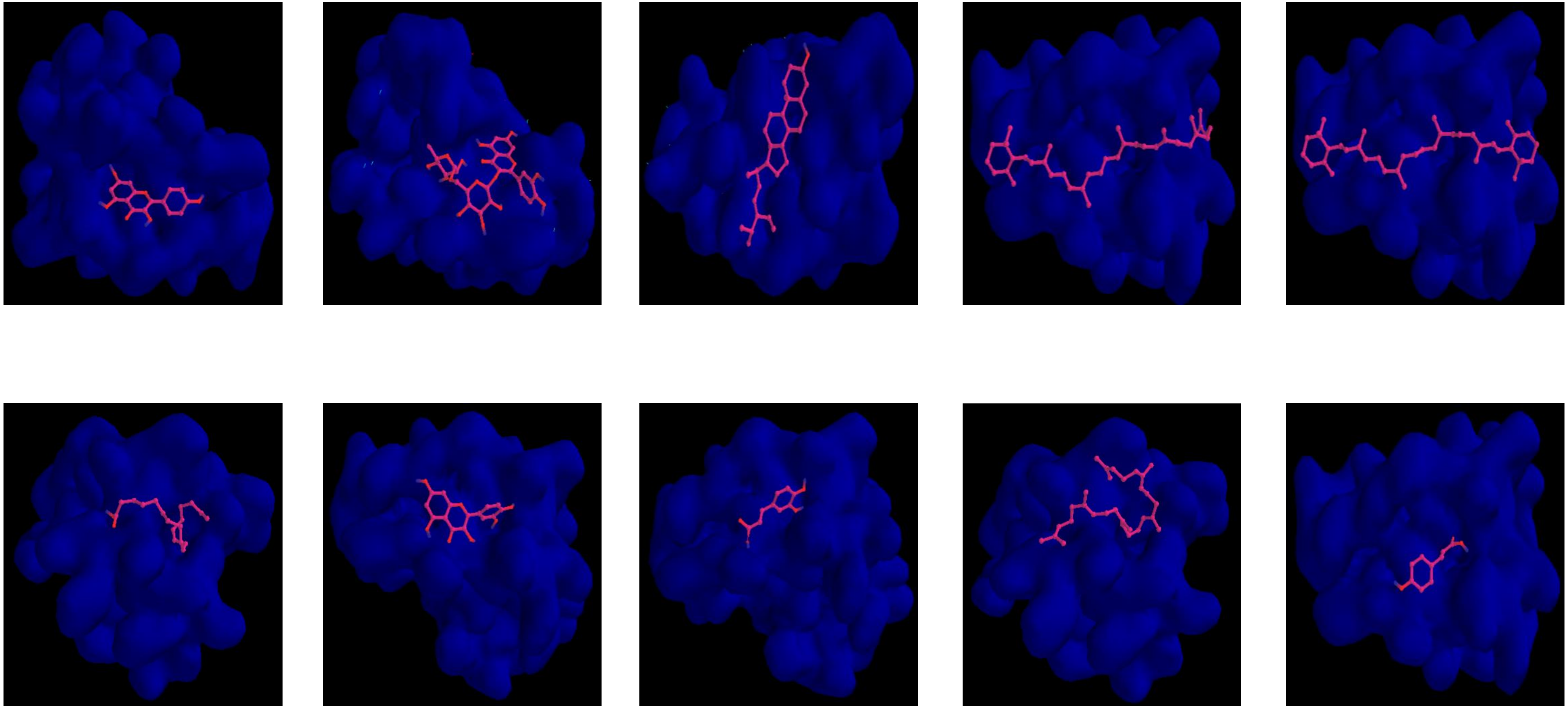

Supplementary Figure S1

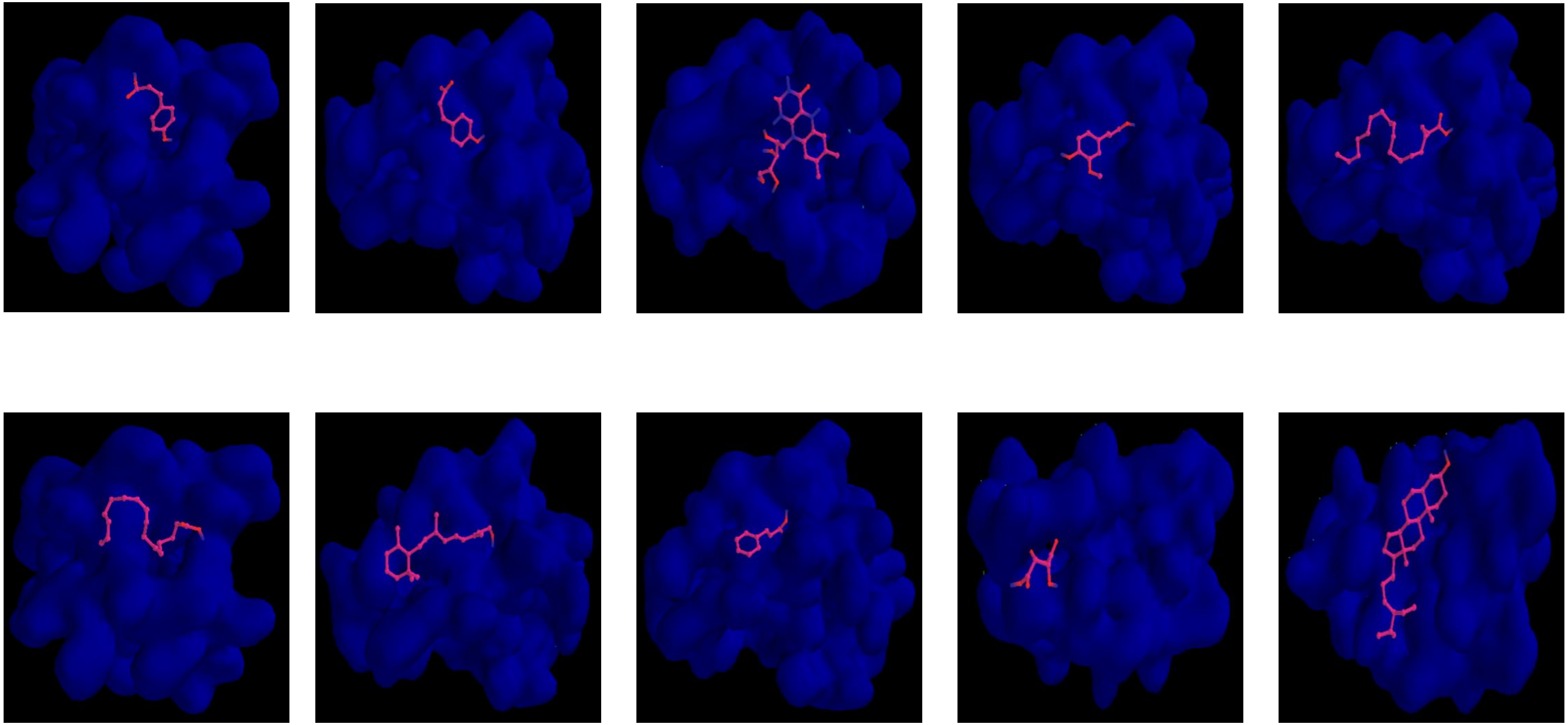

Supplementary Figure S1

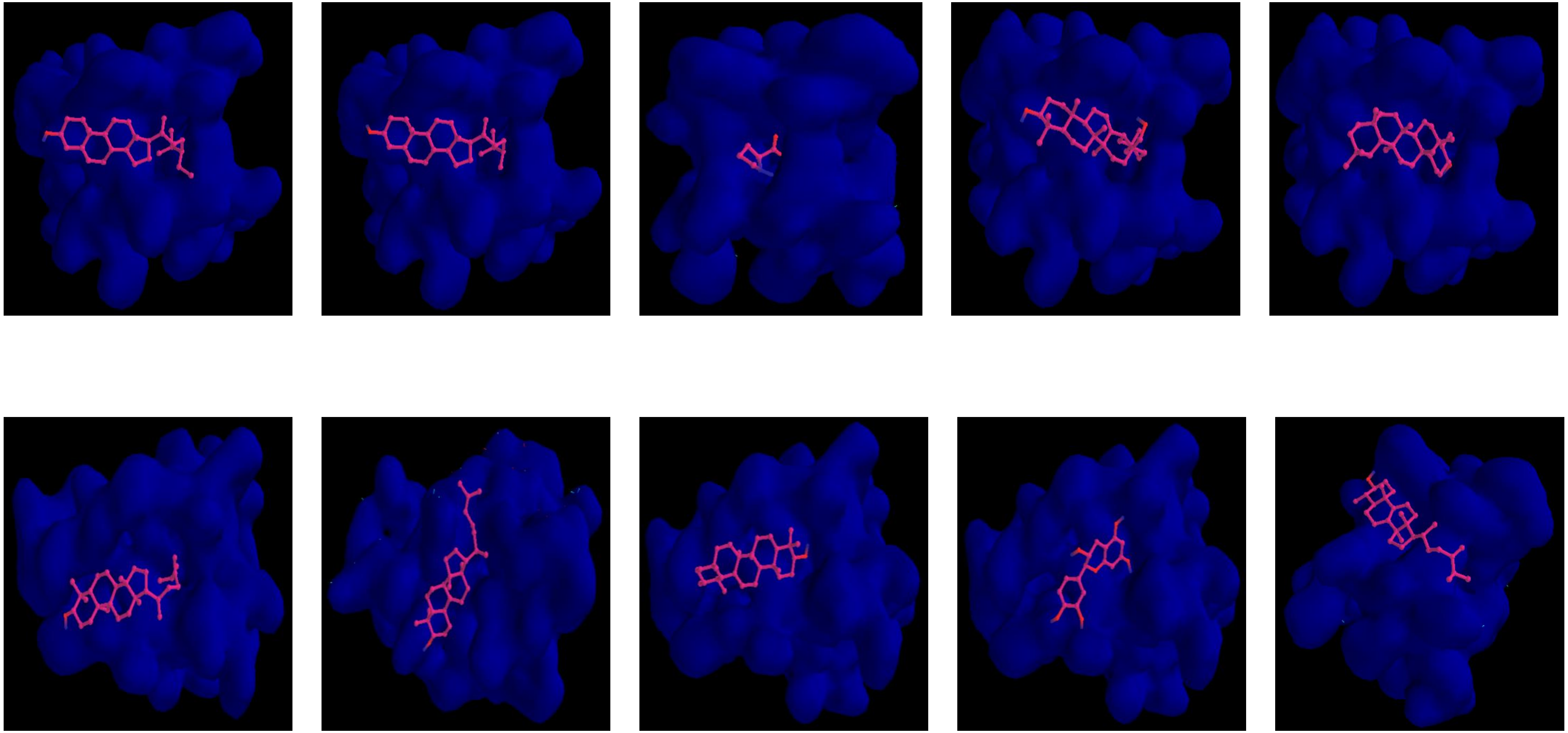

Supplementary Figure S1

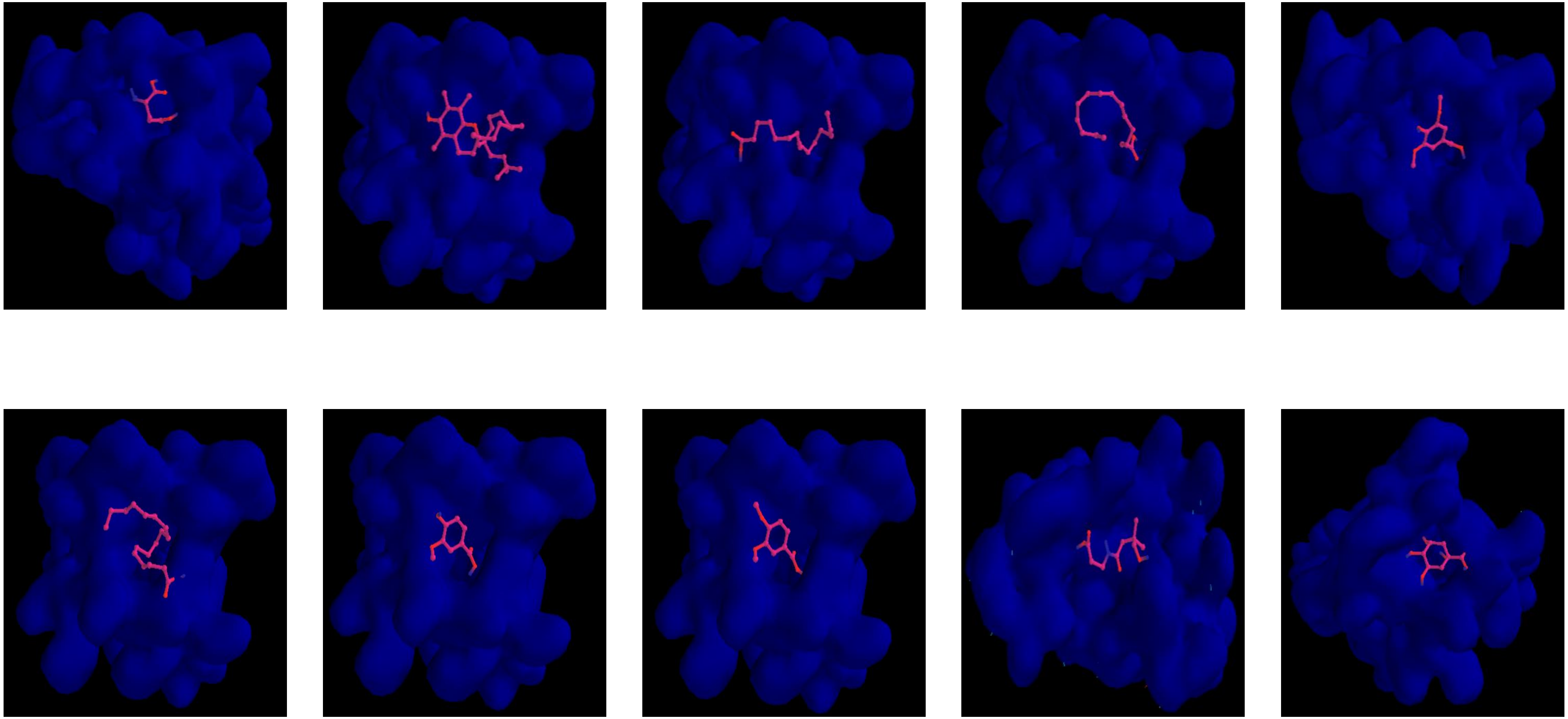

Supplementary Figure S1

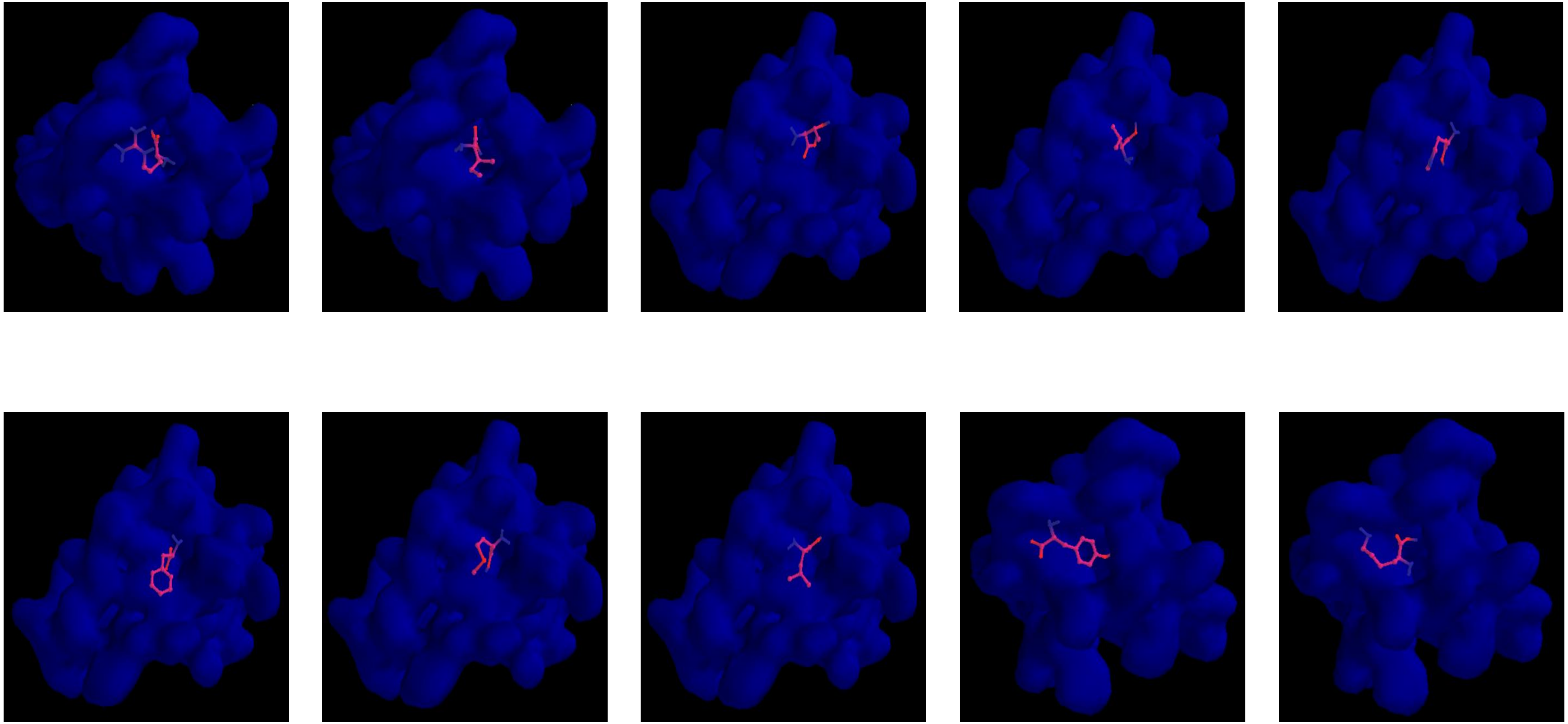

Supplementary Figure S1

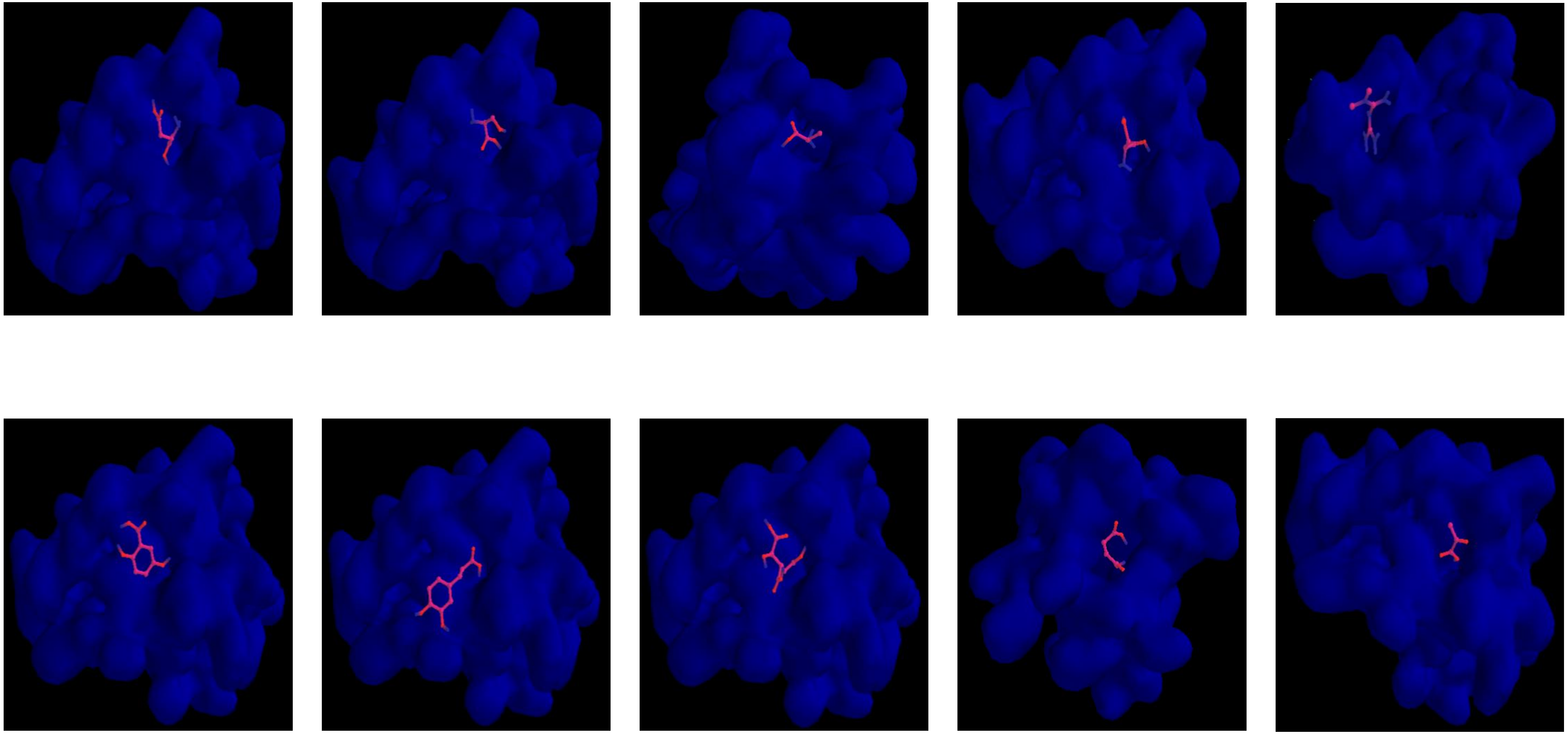

Supplementary Figure S1

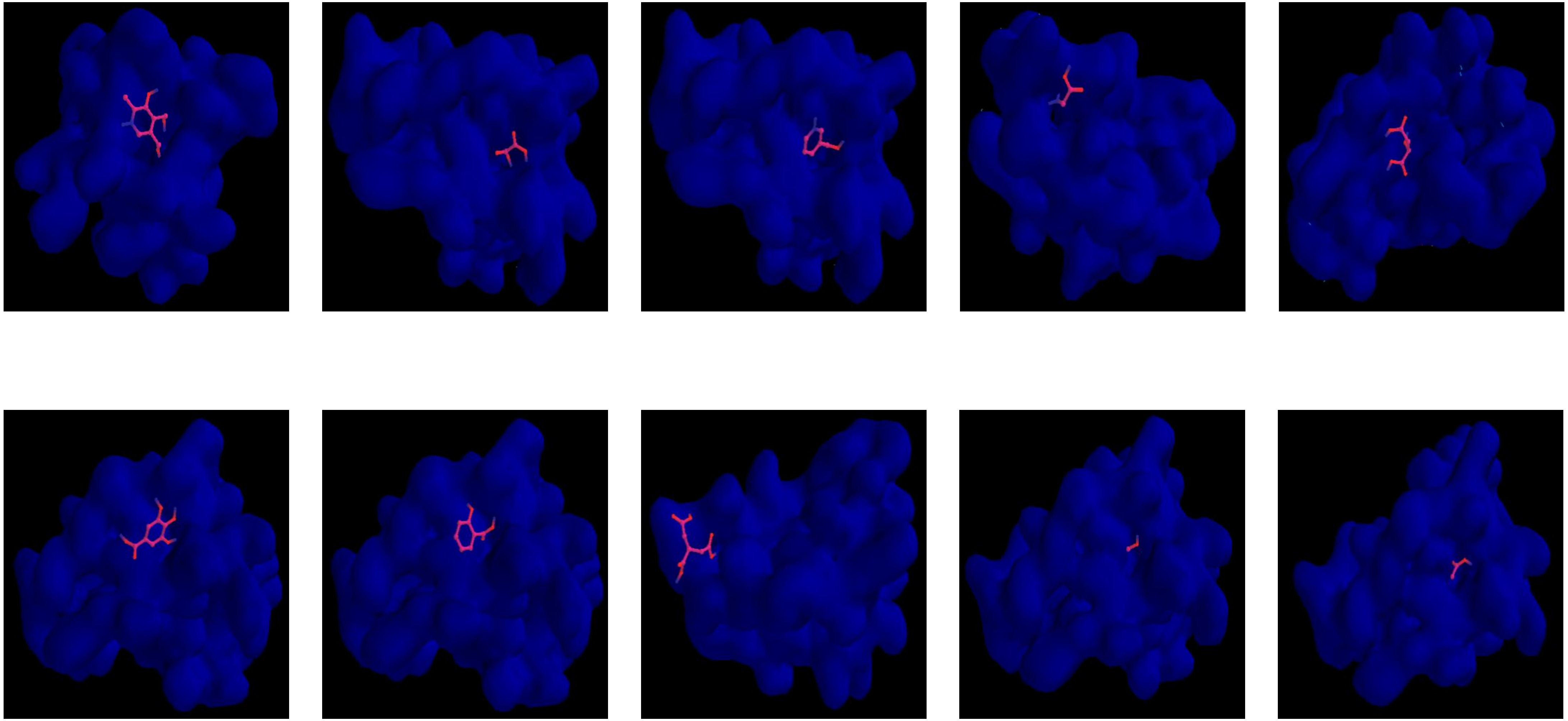

Supplementary Figure S2

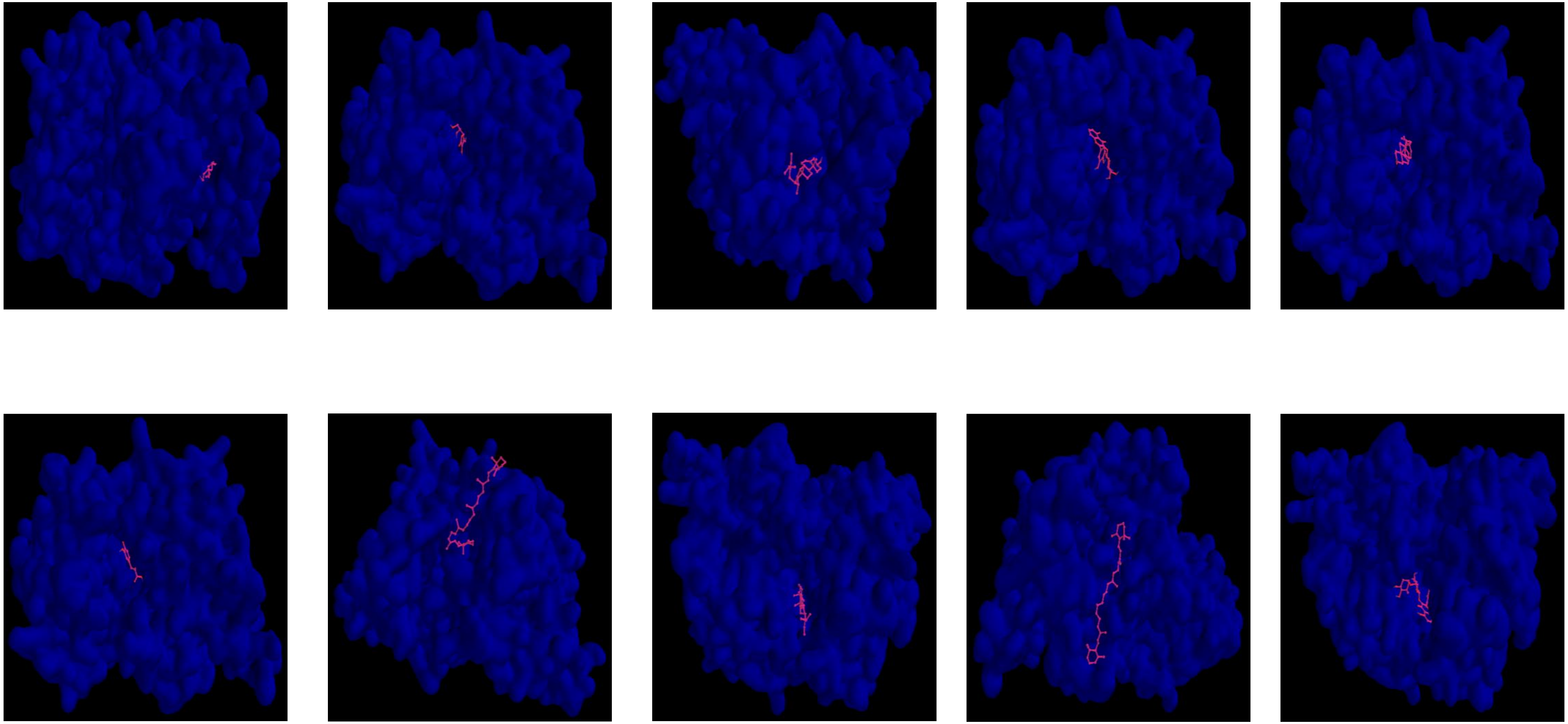

Supplementary Figure S2

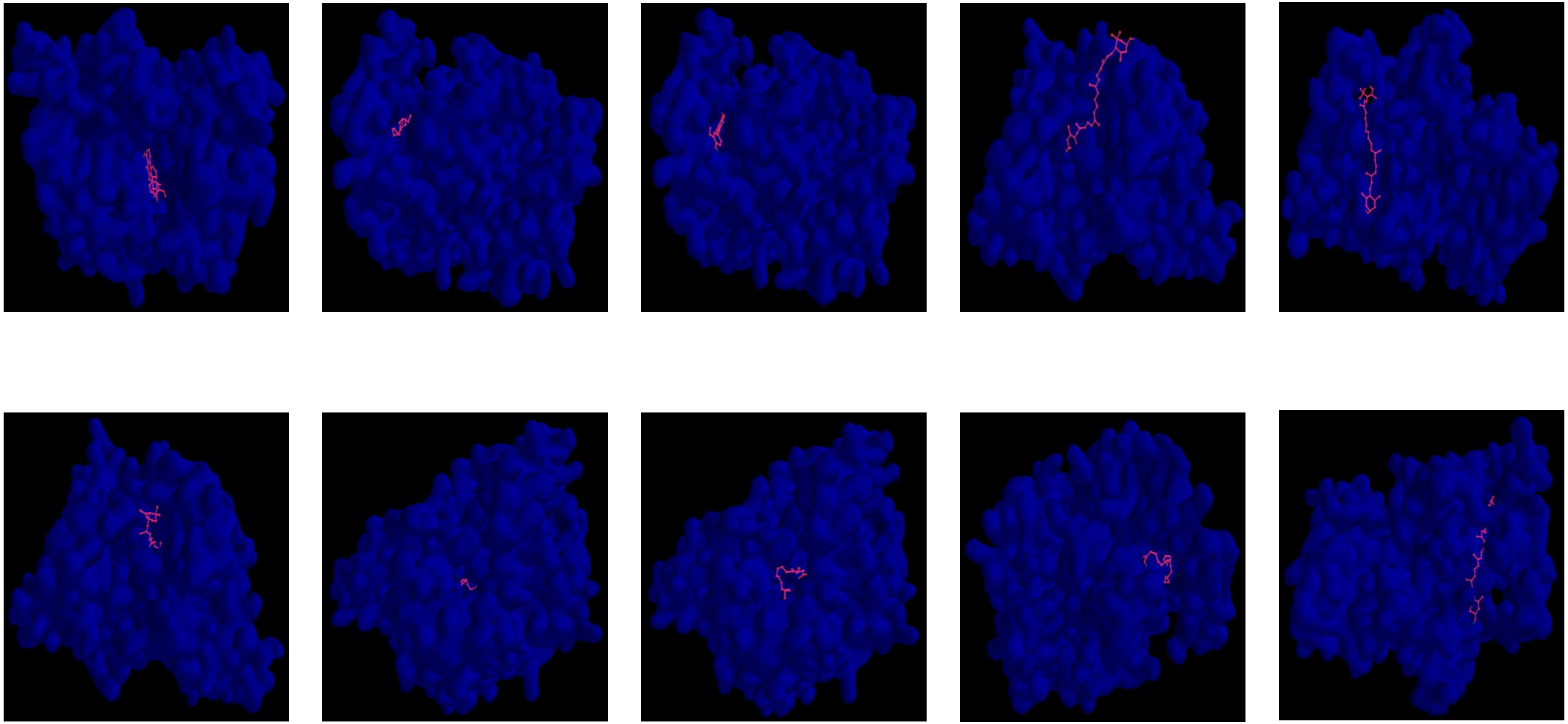

Supplementary Figure S2

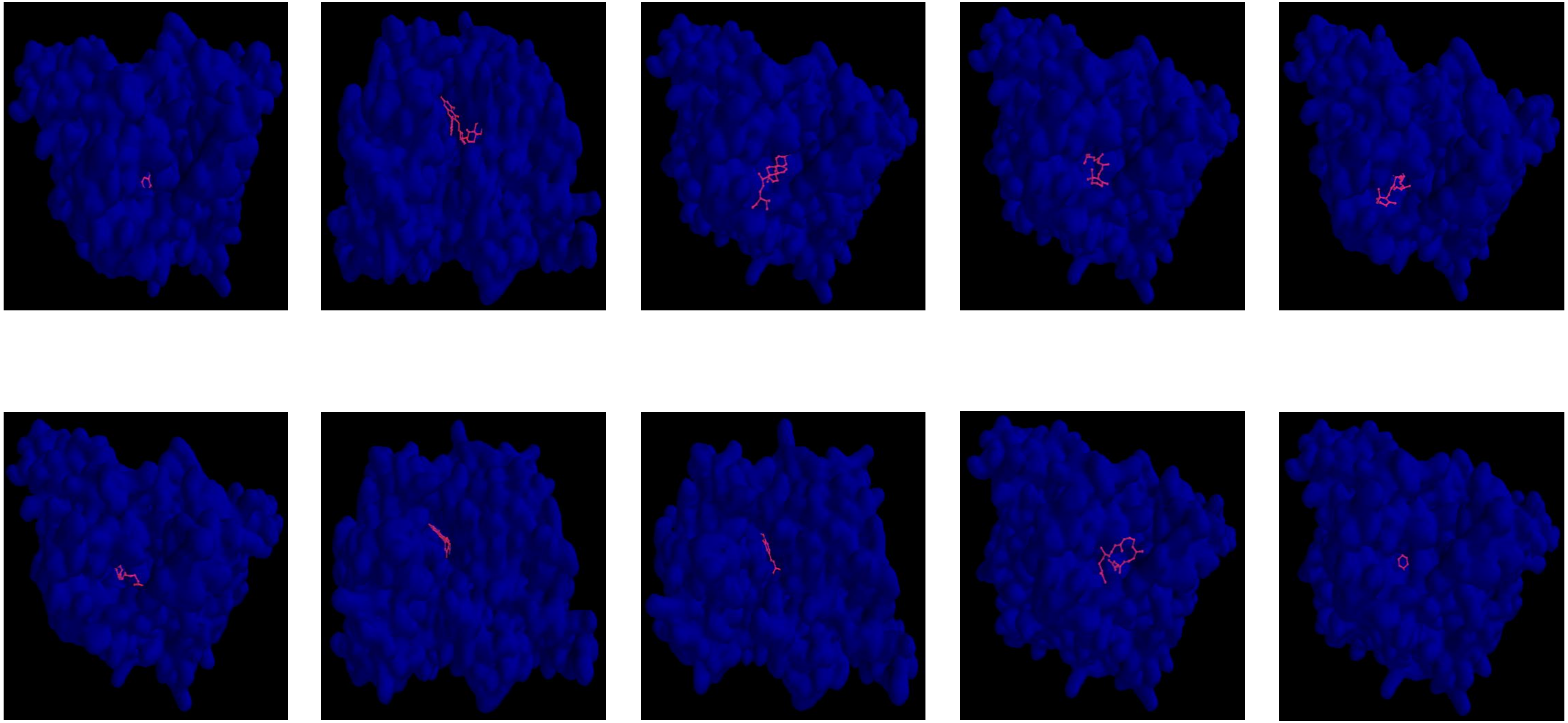

Supplementary Figure S2

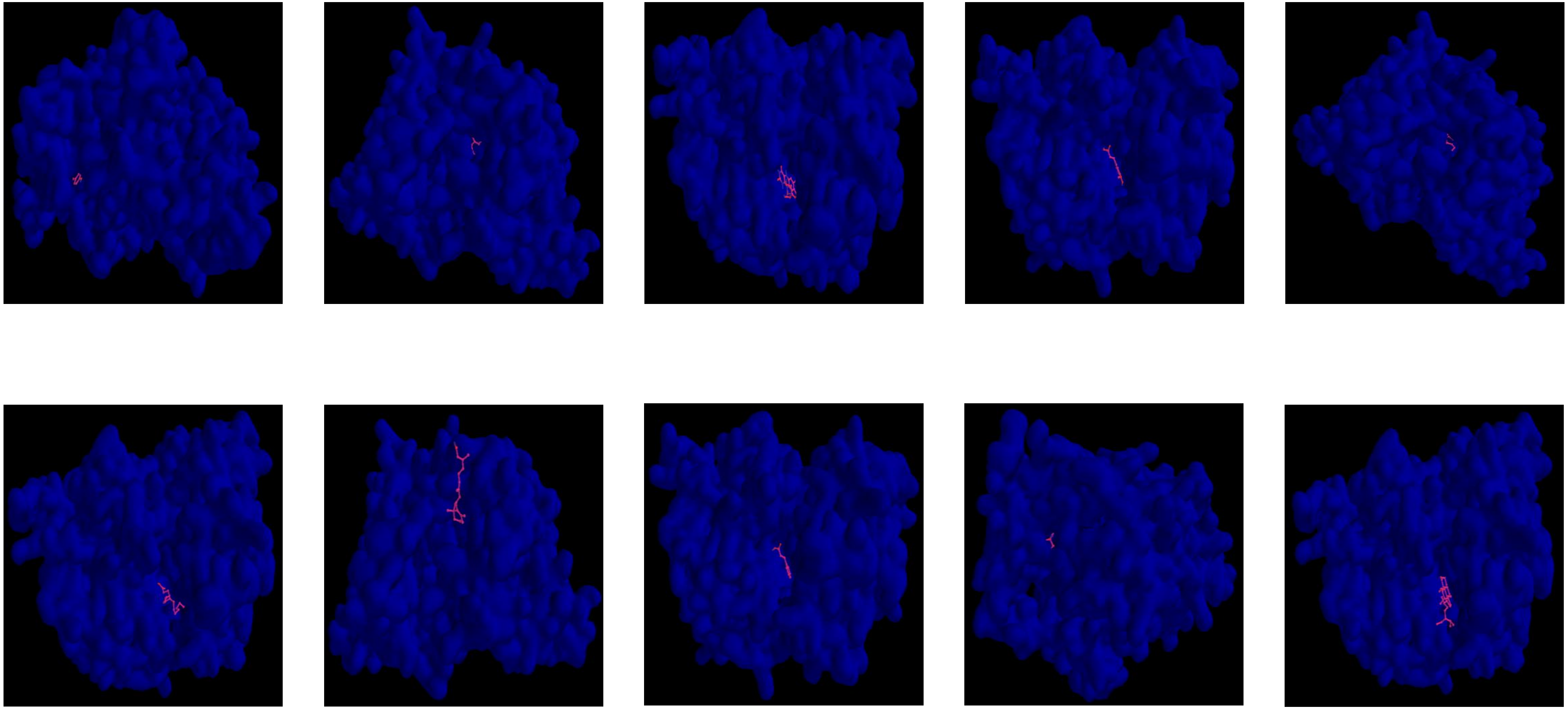

Supplementary Figure S2

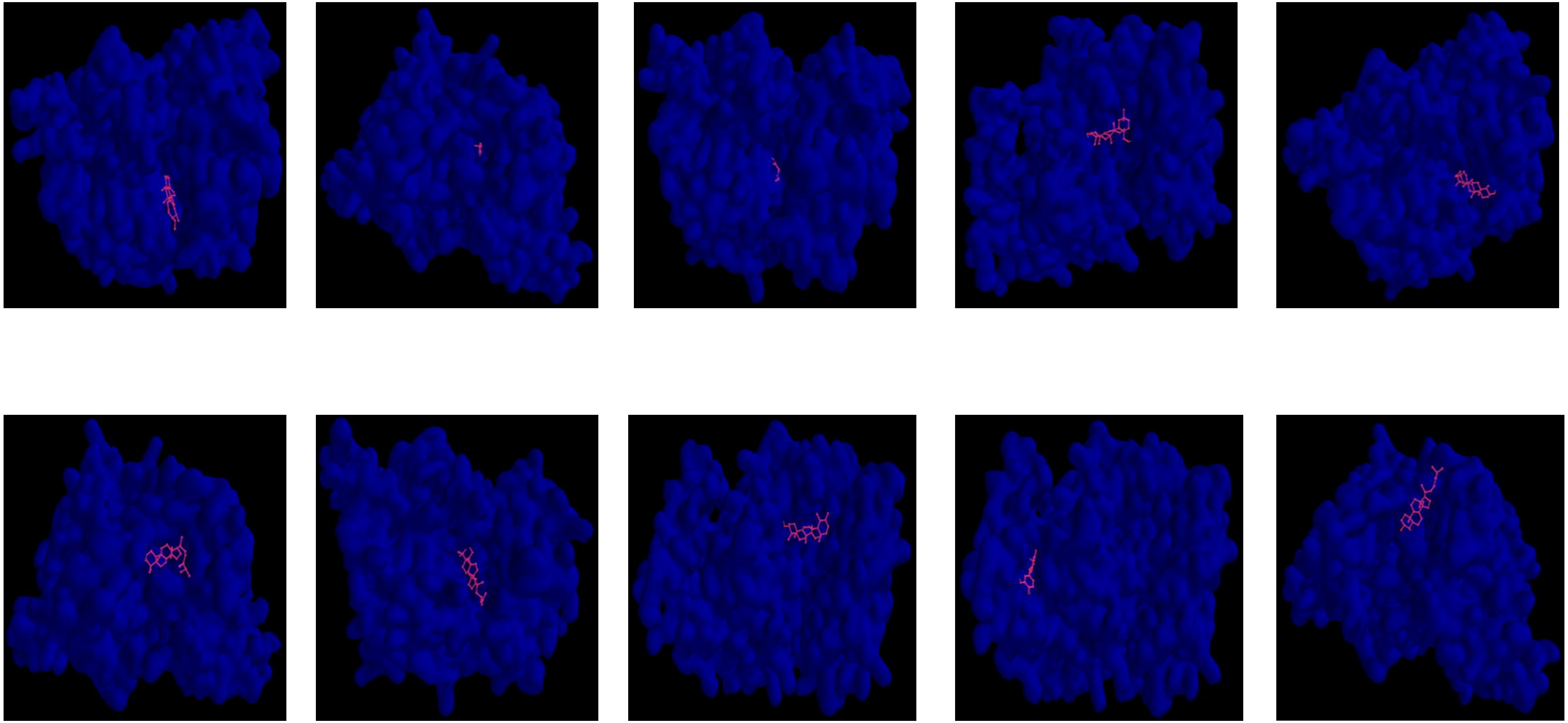

Supplementary Figure S2

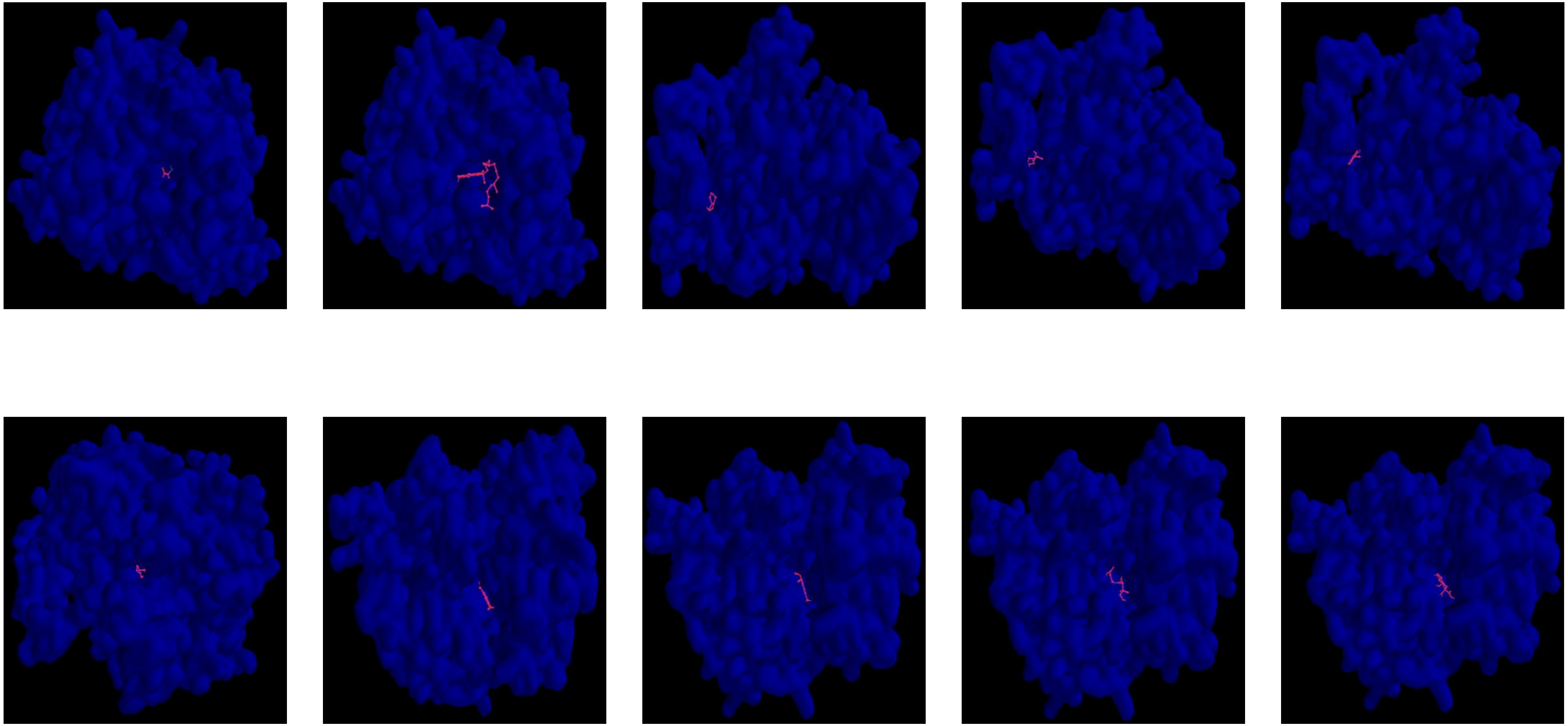

Supplementary Figure S2

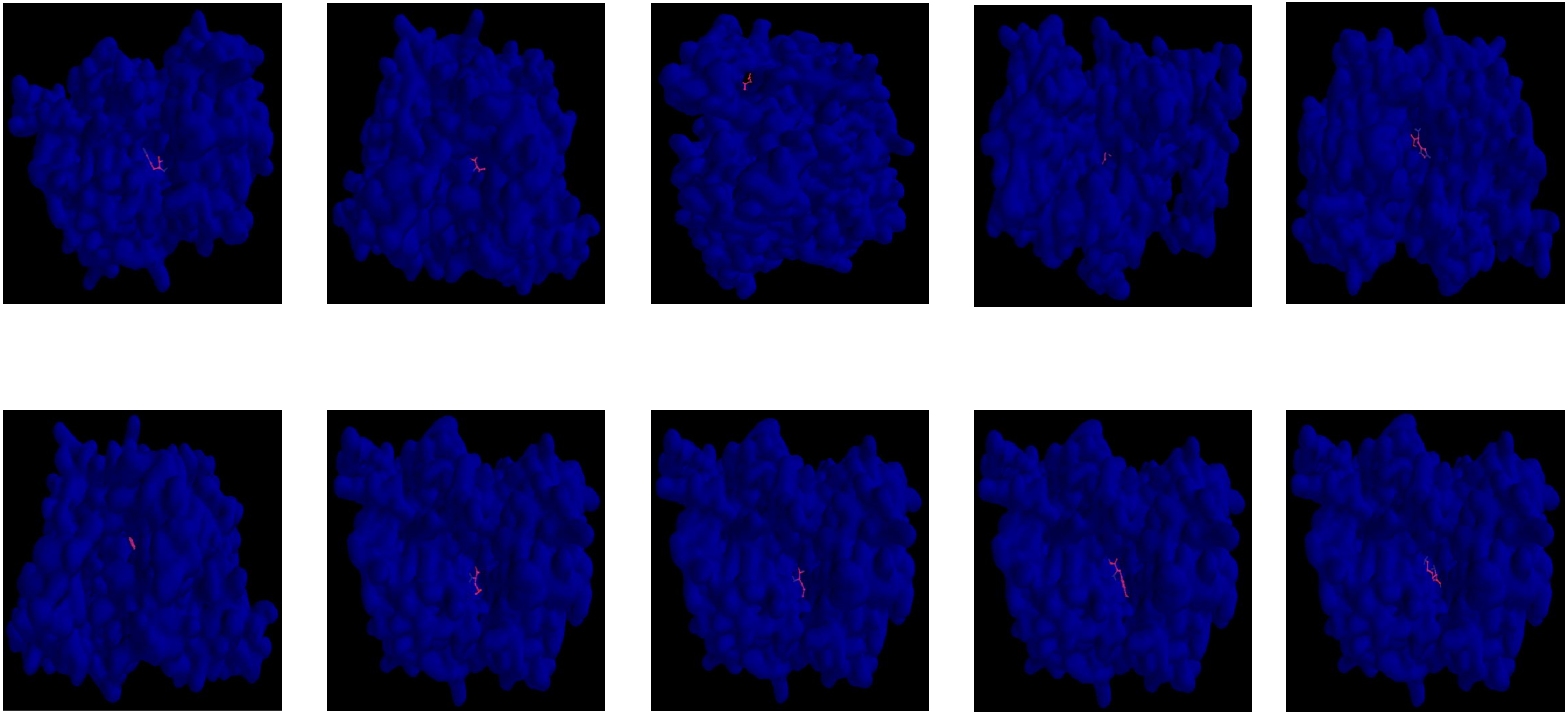

Supplementary Figure S2

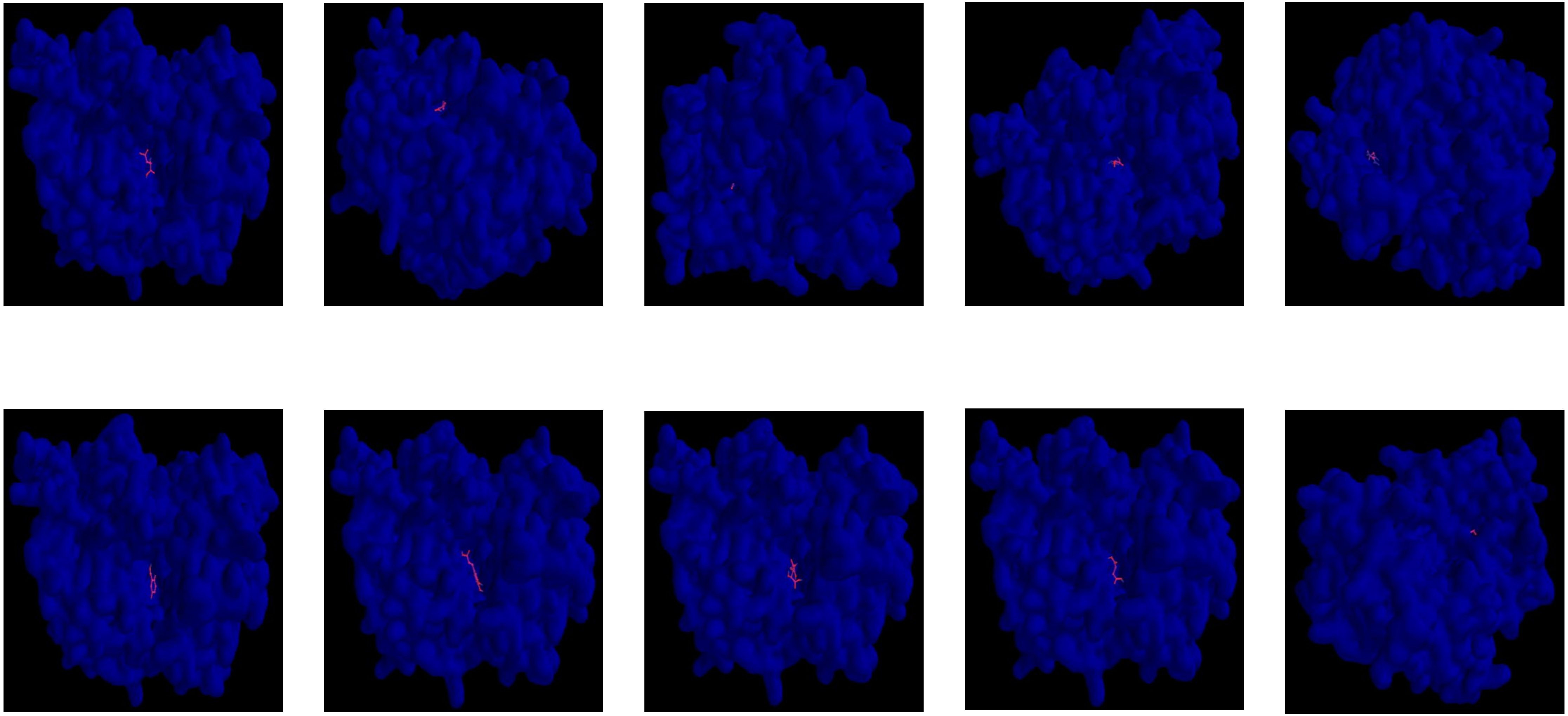

Supplementary Figure S2

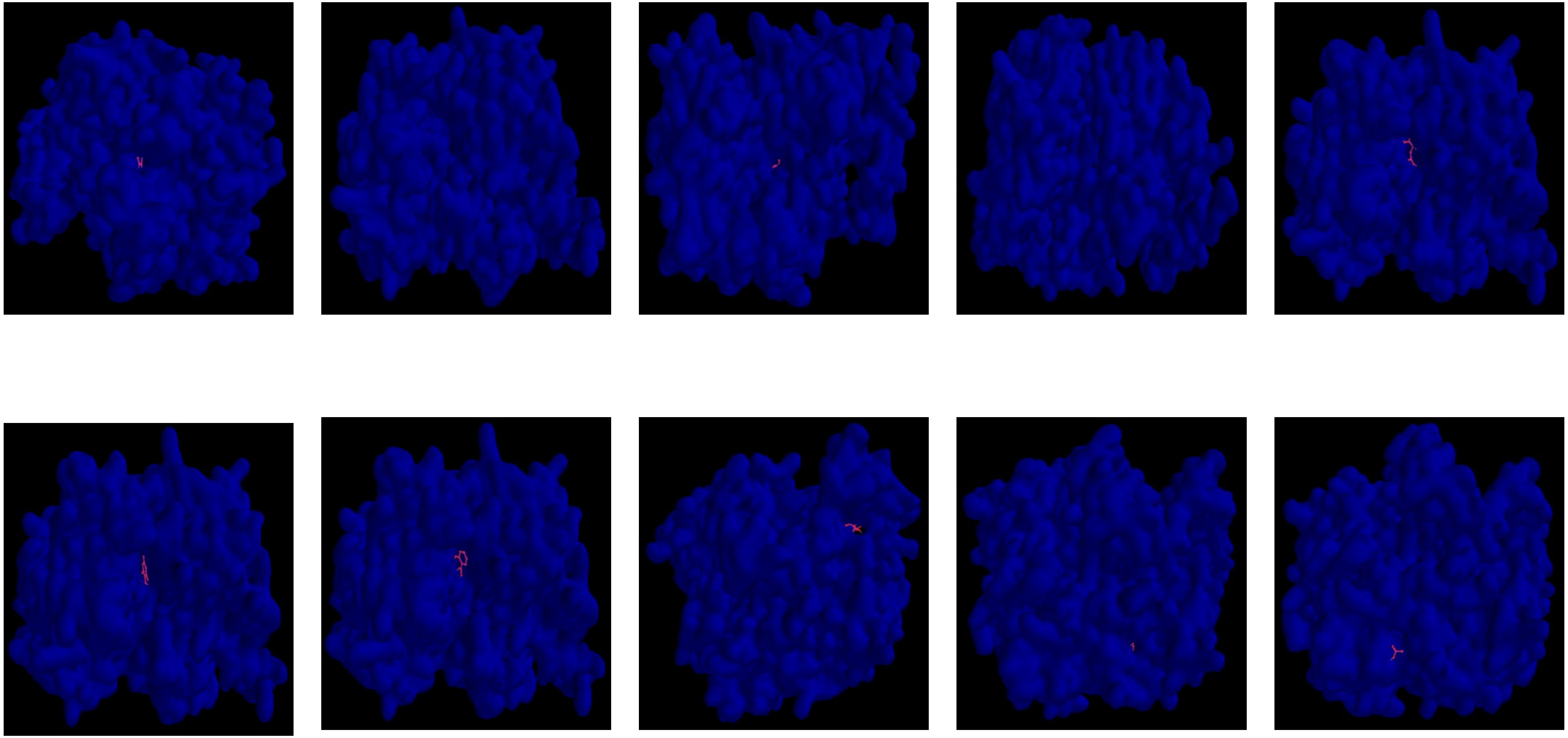

Supplementary Figure S3

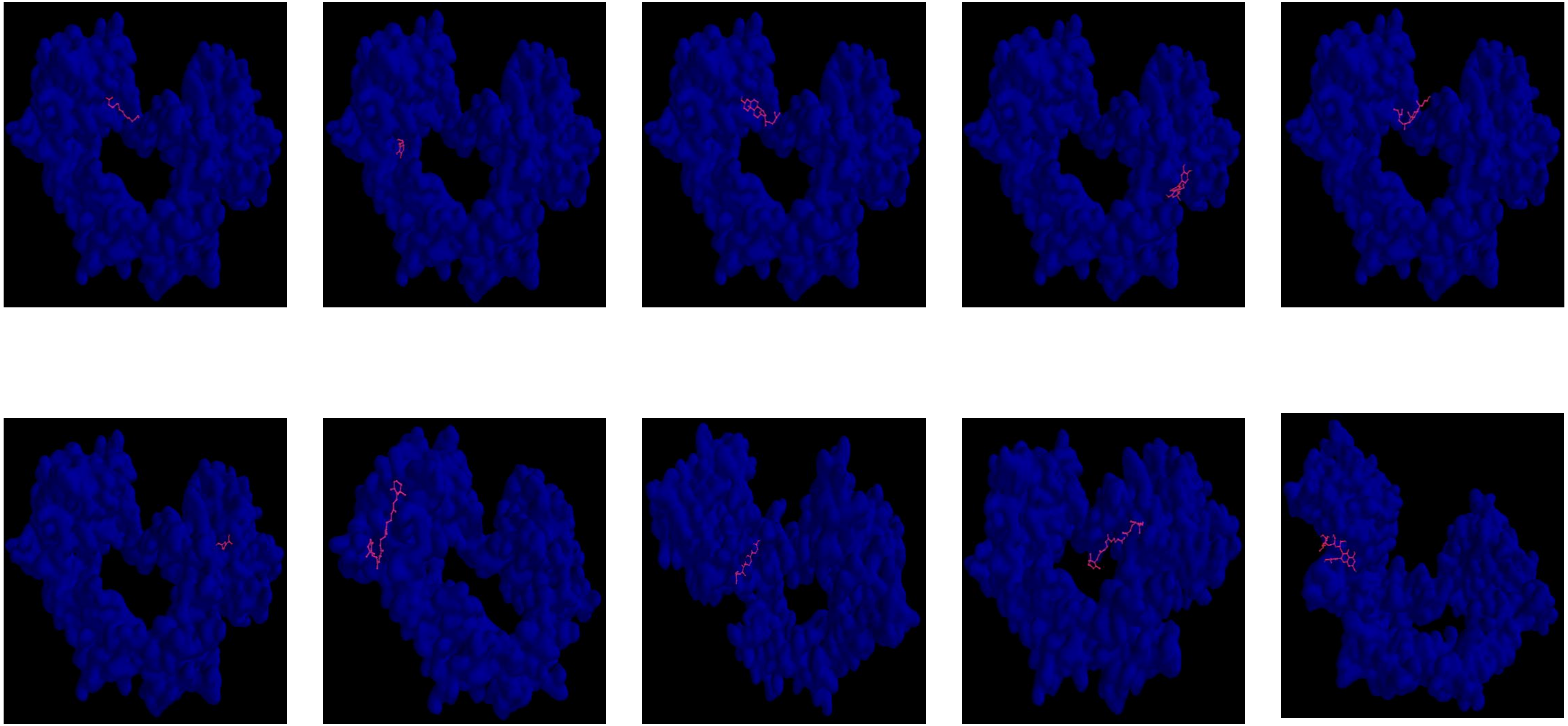

Supplementary Figure S3

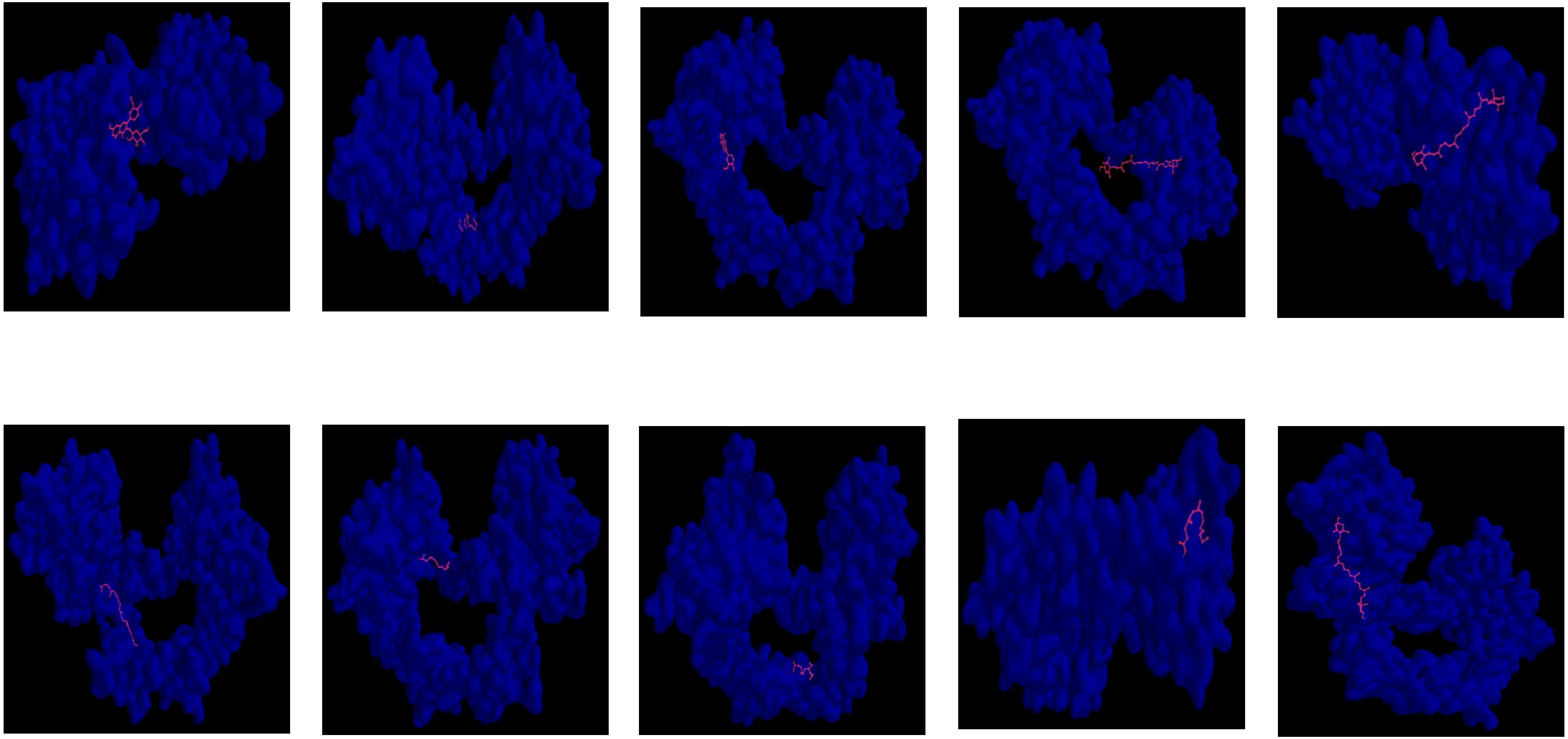

Supplementary Figure S3

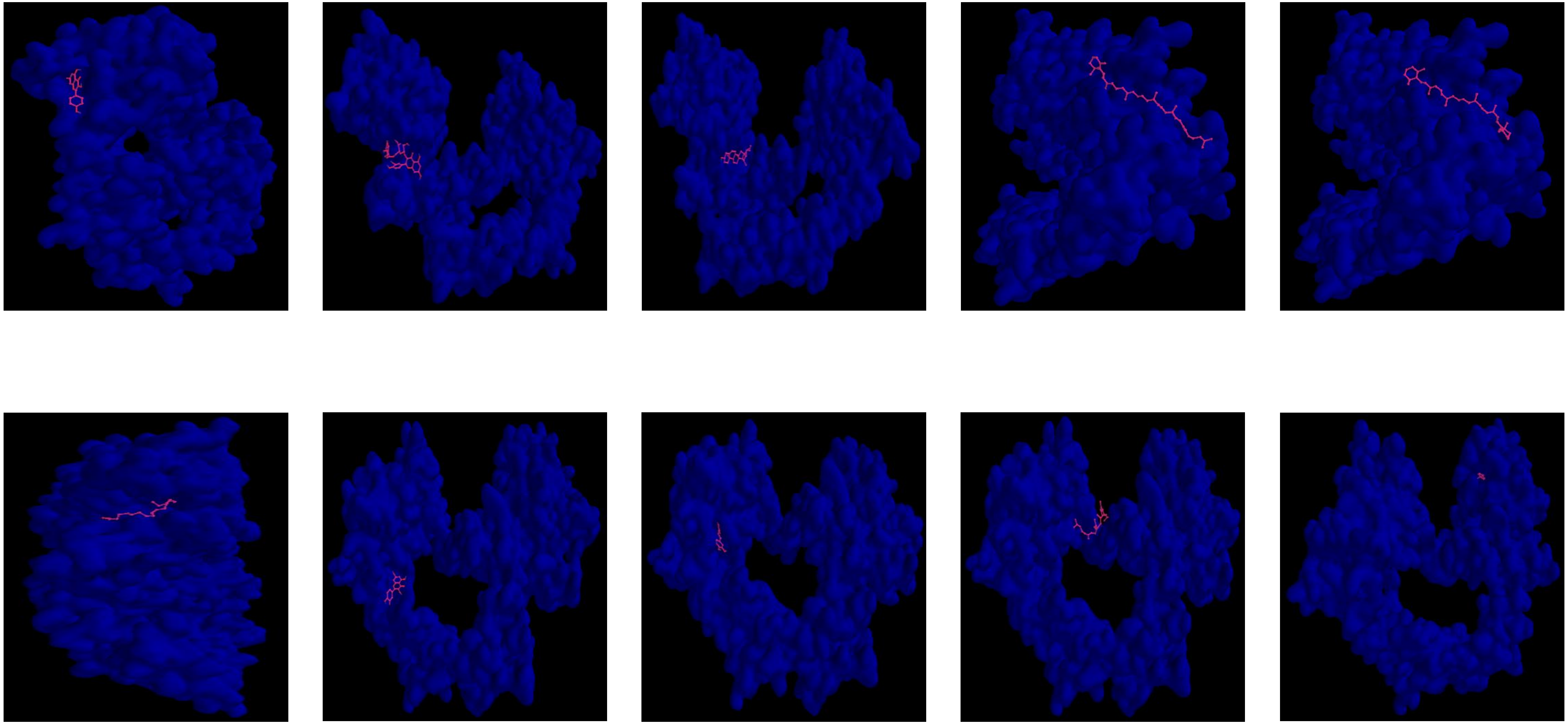

Supplementary Figure S3

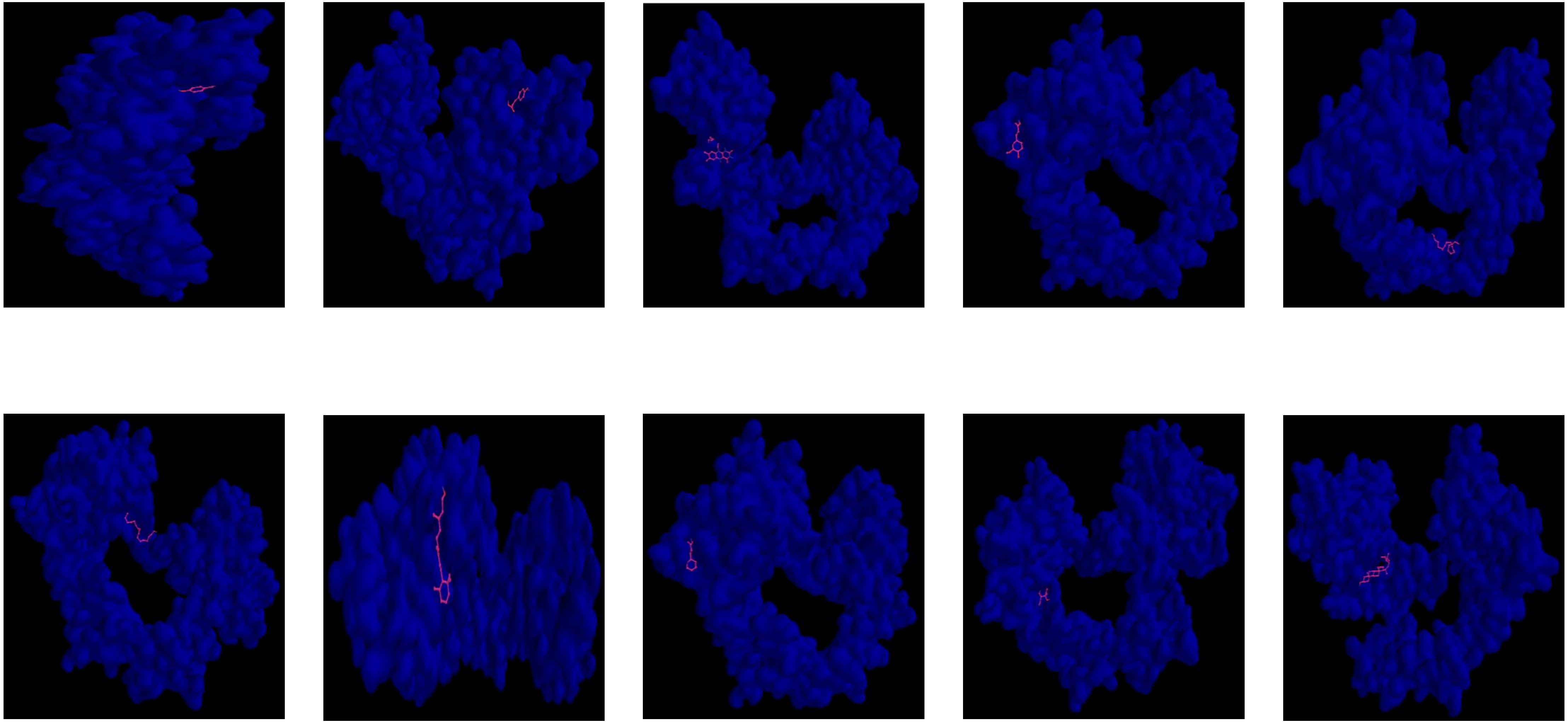

Supplementary Figure S3

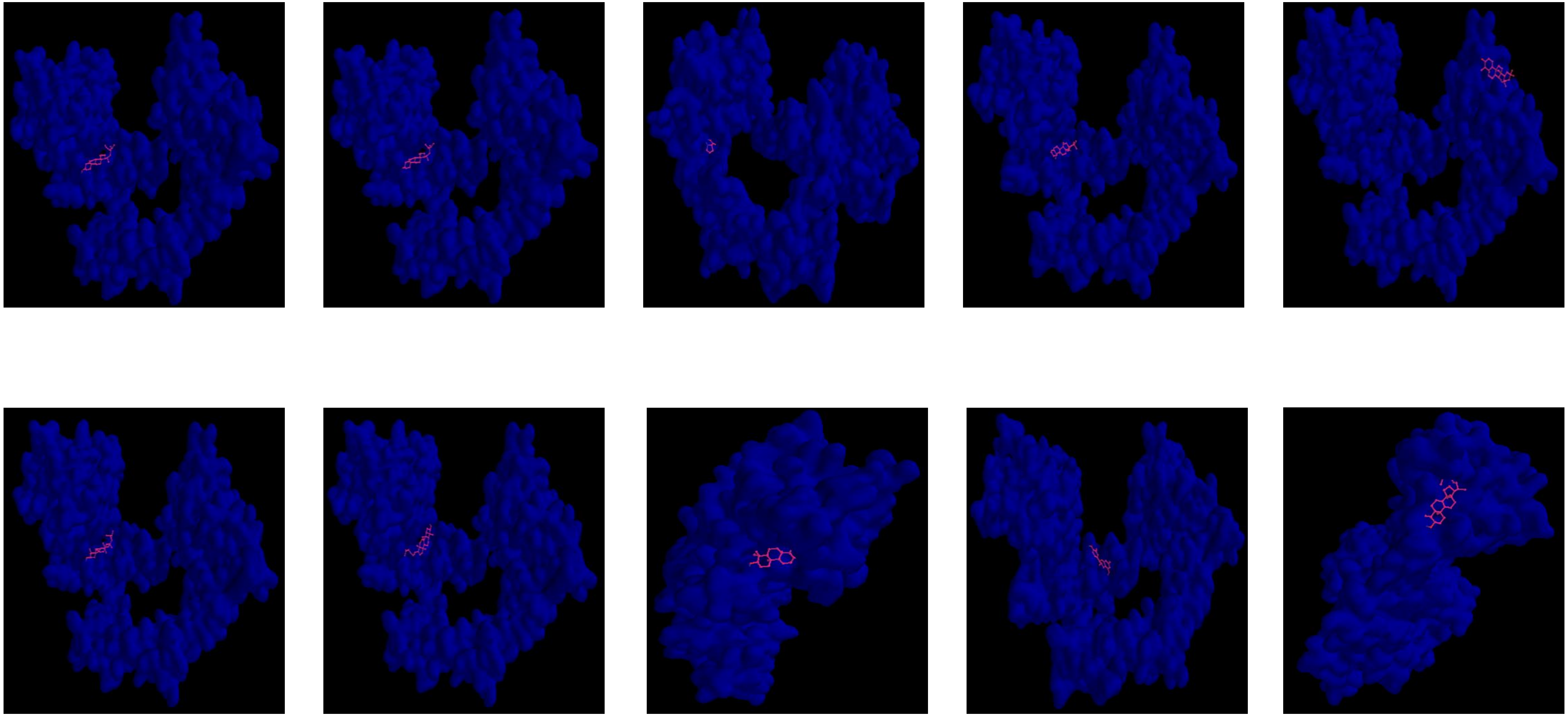

Supplementary Figure S3

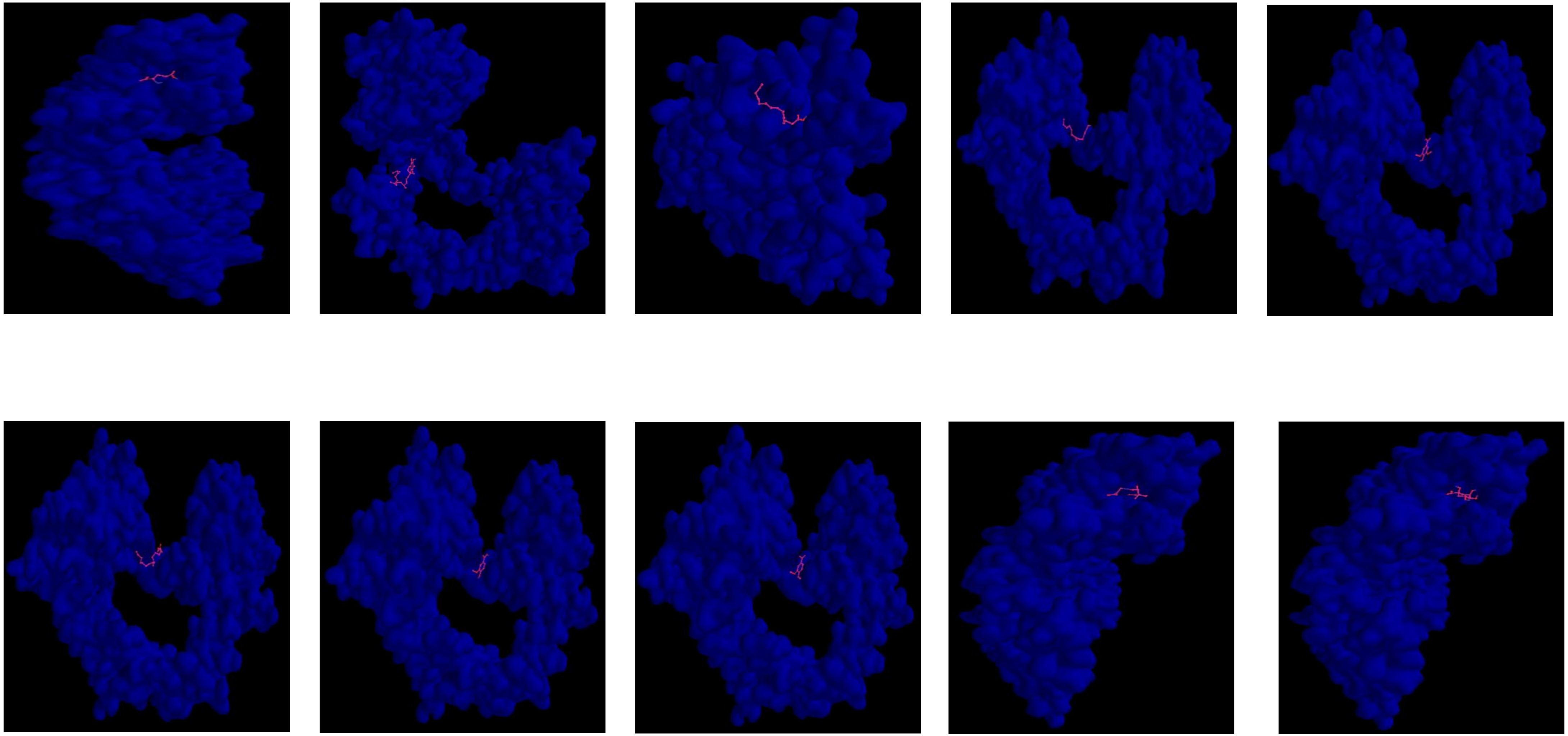

Supplementary Figure S3

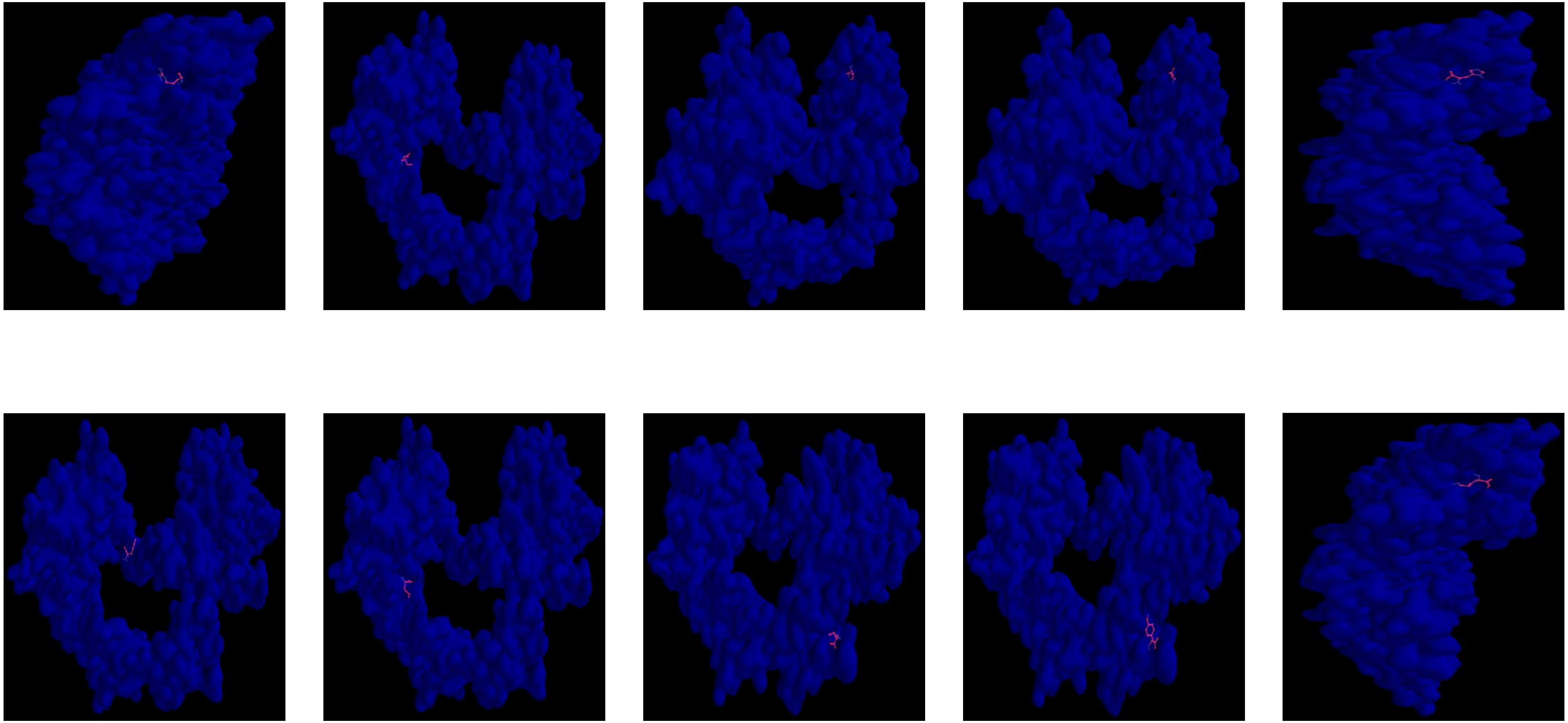

Supplementary Figure S3

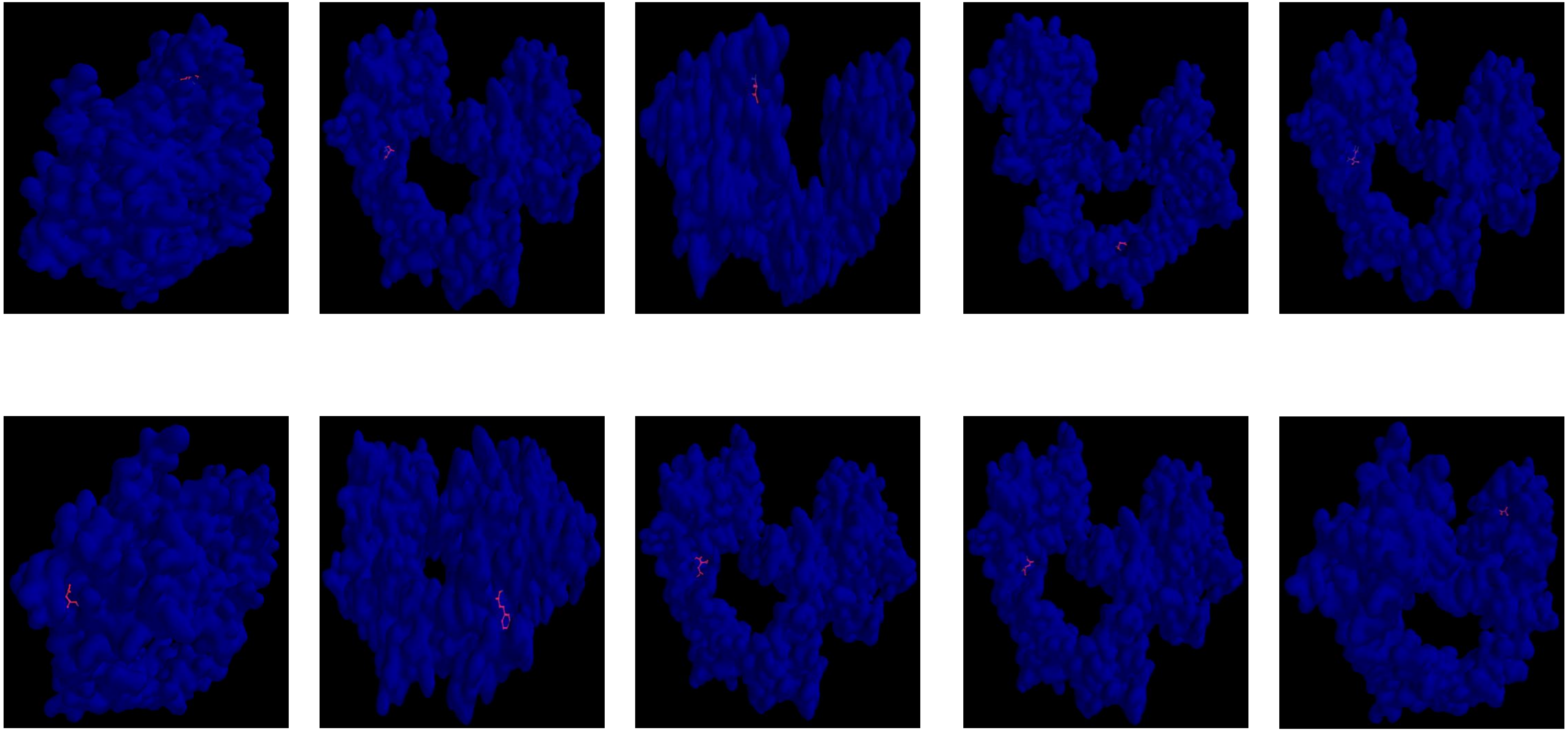

Supplementary Figure S3

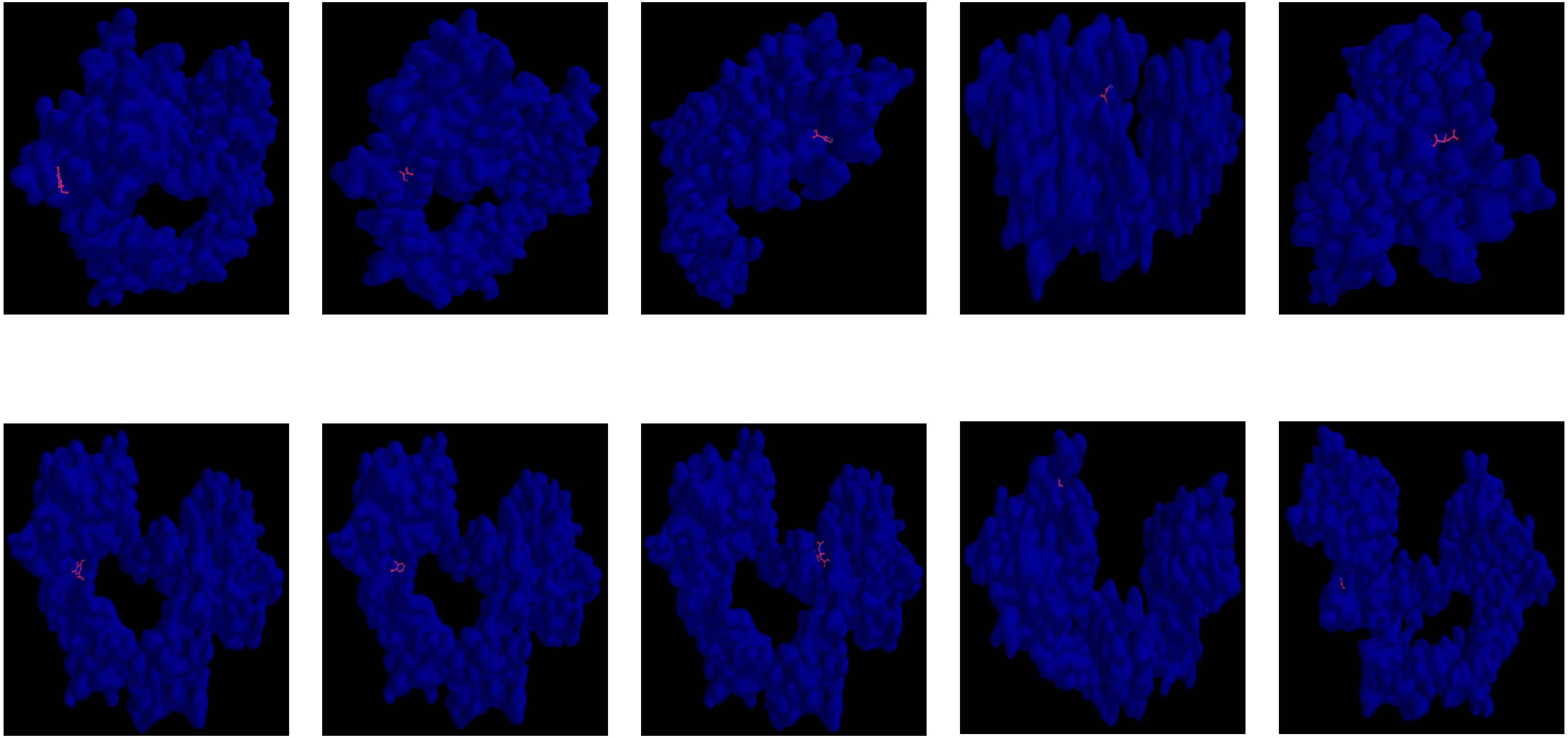

Supplement: Supplementary file 1 [file biology-14-00361-s001.zip › biology-3473425-supplementary.pdf]
